# Effect of Live Environmental Music Therapy and Prerecorded Music on State Anxiety, Stress, Pain, and Well-Being Levels of Patients and Caregivers in the Emergency Department Waiting Room: Protocol for a Multicenter Randomized Clinical Trial

**DOI:** 10.2196/69131

**Published:** 2025-06-18

**Authors:** Angélica Hernández, Ana María Díaz, Ornella Fiorillo Moreno, Raúl Suárez, Moshé Amarillo, Ana María Moreno, Bryan Alonso Ríos Suarez, Lina Marcela Gómez González, Guiselle Alexandra Cristancho Olaya, Mark Ettenberger

**Affiliations:** 1 Clínica Keralty Ibagué Clínica Colsanitas Ibagué, Tolima Colombia; 2 SONO - Centro de Musicoterapia Bogotá Colombia; 3 Clínica Iberoamérica Clínica Colsanitas Barranquilla Colombia; 4 Research Department SONO - Centro de Musicoterapia Bogotá Colombia

**Keywords:** anxiety, emergency department, mental health, music therapy, pain, stress, waiting area

## Abstract

**Background:**

Patients and caregivers attending emergency units often experience elevated levels of stress and anxiety. Music has been used in waiting rooms to reduce stress and anxiety, but existing studies on music therapy in emergency unit waiting areas are scarce and have limitations such as low statistical power and limited music selection.

**Objective:**

The aim of this study is to determine the effect of live Environmental Music Therapy and prerecorded music on state anxiety, stress, pain, and well-being levels in patients and caregivers in the emergency unit waiting areas of 2 hospitals in Colombia.

**Methods:**

This study is a multicenter randomized clinical trial, with 3 arms: standard care + live Environmental Music Therapy, standard care + prerecorded music, and standard care only. The primary outcome measure is the 6-item State-Trait Anxiety Inventory (STAI-6). Secondary outcome measures are pain and stress levels, both measured with Visual Analogue Scales (VAS), and well-being, measured with the Well-Being Numerical Rating Scales (WB-NRSs). The scales will be applied before and after each intervention. A total of 246 patients (82 in each arm) and 147 caregivers (49 in each arm) will be randomized.

**Results:**

Data collection for this study started on September 3, 2024, and as of the submission of the study (November 2024), 216 patients and 134 caregivers have been enrolled. It is expected that the results will be available by May 2025.

**Conclusions:**

This study seeks to contribute to improving the mental health, well-being, and quality of care of patients and caregivers in the waiting area of the emergency units. This is the first study in Colombia investigating the effect of live music therapy and prerecorded music interventions in the emergency department.

**Trial Registration:**

ClinicalTrials.gov NCT06510153; https://clinicaltrials.gov/study/NCT06510153

**International Registered Report Identifier (IRRID):**

DERR1-10.2196/69131

## Introduction

Emergency departments (ED) are critical for providing immediate care to patients with acute illnesses or those with medical emergencies. Its proper functioning depends on an organized triage system, which guarantees correct prioritization according to the urgency of pathologies [1]. Emergency health care professionals face a wide variety of medical needs and are exposed to complex social interactions with patients, family members, and caregivers. As a result, the environment in ED waiting rooms can be challenging and stressful for both patients and staff [2-5]. Distress may result from patients’ exposure to pain, injuries, discomfort, and possible diagnosis or medical treatments, which can increase their levels of anxiety and the feeling of uncertainty in the face of often prolonged waiting times [6]. Long waiting times in the ED have been shown to negatively affect patient recovery and patients’ and caregivers’ perception of the care quality provided [7-9]. In addition, patients can also experience nausea, hunger, or thirst, which, as they are not as easily detected by the staff as pain or dyspnea for example, can increase anxiety, insecurity, and discomfort in the waiting area [10]. Thus, it is important to consider environmental factors of ED waiting rooms, such as the quality of chairs, lighting, screens, and sounds, as they also affect the emotional states of patients and caregivers [11].

To mitigate some of these challenges, music therapy and other music-based interventions are increasingly applied in EDs. Early publications include reports of nursing teams using music in the pediatric emergency ward to reduce behaviors such as crying and screaming in children [12,13]. More recent studies highlight the benefits of prerecorded music in reducing anxiety [14-16] or pain [17] levels in adult patients in ED observation rooms. A study on live music therapy in emergency rooms was conducted by Mandel et al [18], including 180 patients (90 intervention, 90 control) who received music therapy sessions over a 3-year period. Although no statistically significant differences were found in patient satisfaction, stress, and pain levels decreased significantly in those patients who received music therapy. Furthermore, staff reported that music therapy favored the overall care experience and patients expressed a desire to have music therapy intervention in the ED again [18].

With respect to waiting rooms, recent literature reviews confirm the potential benefits of music in these settings but also highlight the methodological heterogeneity of studies, small sample sizes, limited descriptions of the music used, and a lack of live music approaches [19,20]. One of the few studies reporting live approaches was conducted by Van Dokkum et al [21] and Rossetti et al [22]. Van Dokkum et al [21] investigated the impact of visiting artists performing live music in waiting rooms at a hospital in New York, showing a positive impact on the sound environment. In a randomized controlled trial, Rossetti et al [22] investigated the effect of live Environmental Music Therapy (EMT) on anxiety levels and waiting time perception of patients and caregivers in the radiation therapy waiting room. The results showed a significant reduction in stress and anxiety levels in both patients and caregivers, in addition to shortening the perception of waiting time.

This study protocol seeks to expand the current knowledge on the use of music in ED waiting rooms by determining the effect of standard care + live EMT versus standard care + prerecorded music versus standard care alone on state anxiety, stress, pain, and well-being levels of in adult patients and caregivers in the emergency waiting rooms of the hospitals Clínica Keralty Ibagué and Clínica Iberoamérica in Colombia, South America.

## Methods

### Study Design

This study follows the SPIRIT (Standard Protocol Items: Recommendations for Interventional Trials) guidelines (Multimedia Appendix 1) for reporting randomized clinical trial (RCT) protocols [23] and is a multicenter, pragmatic, RCT with 3 parallel arms [24]:

- Intervention group 1: Standard care + EMT
- Intervention group 2: Standard care + prerecorded music
- Control group: Standard care alone

This study will be carried out in the emergency waiting room areas of the Clínica Keralty Ibagué and Clínica Iberoamérica within a 6-month period. The anticipated recruitment start date is September 2024.

### Study Participants and Setting

The study participants are adult patients and caregivers in the emergency waiting rooms of both hospitals. Both EDs are located in different cities in the country but belong to the same health care provider, which ensures similar standard care procedures for patients and caregivers. Both EDs serve subsidized, contributory, and prepaid health care users and both hospitals use the ESI (Emergency Severity Index) system for effective triage of patients. The ED waiting area at the Clínica Keralty Ibagué has 40 seats and the one at the Clínica Iberoamérica has 38 seats. Estimated wait times according to triage classification are as follows: triage 1: immediate attention (reanimation); triage 2: approximately 30 minutes; triage 3: approximately 1 hour; and triage 4 and 5: up to 6 hours. The 3 most common reasons for admission to the ED are (1) abdominal and pelvic pain, (2) fever of unknown origin, and (3) intestinal infections due to viruses and other pathogens.

#### Inclusion Criteria

The inclusion criteria are patients and caregivers of legal age present in the ED waiting rooms during the intervention and control conditions; patients whose triage does not represent an immediate threat to life or severely ill patients (ie, triage 3, 4, and 5); and patients and caregivers with the necessary literacy and cognitive skills to understand and fill out the questionnaires.

#### Exclusion Criteria

The exclusion criteria are patients and caregivers who do not consent to participate in the study; and patients and caregivers who report having hearing problems.

#### Sample Size

The sample size was calculated based on the primary outcome of this study using G*Power 3.1.9.7 (Heinrich Heine University Düsseldorf) [25] and considering that the study design corresponds to a 3×2 factorial design (3 groups and 2 repeated measures). Therefore, we will determine the effect of treatment between the groups using a 2-way ANOVA and repeated measures. We chose a statistical power of .80, a significance level of .05, and an effect size of =.01, the effect size was determined based on previous research [22] and the expected magnitude of differences between groups. The sample size calculation resulted in 246 participating patients, distributed in 82 per group.

In addition, a separate sample size calculation was performed for participating caregivers, following the same procedure as described for the patients and considering an effect size of η^2^=.017, resulting in 147 caregivers (49 per group). Both sample sizes are within the range reported in previous studies [22].

#### Participant Selection and Randomization

Selection of the participants will be made by convenience sampling. The intervention and control conditions will take place on the same day of the week (Tuesdays, Thursdays, and Fridays) and at the same hour (11 to 11:30 AM) at both hospitals. The order of the 3 conditions (EMT, prerecorded, control) will be randomized by days of intervention. For this, a block size randomization of multiples of 3 will be used, ensuring an equal number of interventions for each arm of the study. Intervention A will be established as EMT, intervention B as prerecorded music, and intervention C as standard care.

#### Blinding

Due to the nature of the interventions, music therapists, patients, caregivers, and data collectors, will not be blinded. Data analysis will be carried out by a member of the research team blinded to the result of the randomization.

#### Concealment

The concealment of the random assignment will be guaranteed using sealed, sequentially numbered, and opaque envelopes. The envelopes will be sealed with tamper-proof security tape and opened sequentially on each day of intervention. The envelopes will be kept in a folder that will be kept on a locked shelf inside each clinic, which will be in custody by the research assistant at each participating clinic. The envelope will be opened 10 minutes before the start of the intervention to give the music therapists time to organize their equipment and instruments.

#### Primary Outcome Measure

The primary outcome measure is state anxiety, measured with the Spanish version of the 6-item State-Trait Anxiety Inventory (STAI-6). In its original version, the STAI consists of 40 items, of which 20 measure trait anxiety and 20 items measure state anxiety [26]. The STAI-6 is a short version of the state scale and consists of 6 items evaluated on a 4-item Likert scale. The STAI-6 was validated in its original English version by Chlan et al [27], and its Spanish version by Perpiñá-Galvañ et al [28,29], and has been successfully used in a previous study on EMT in radiotherapy waiting rooms [22]. The STAI-6 will be applied before and after each intervention.

#### Secondary Outcome Measures

- Stress: Stress levels will be measured by the Visual Analogue Stress Scale (VAS-S). The VAS-S consists of a 10-cm line divided into 10 points, each separated by 1 cm. 0 means the complete absence of stress, and 10 is the maximum possible stress. The VAS-S has been validated by Lesage et al [30] and was recently applied in a study on stress levels of caregivers or companions in the ED [31]. A cutoff of 7 points has been established as a significant stress level [32]. The VAS-S will apply before and after each intervention.
- Pain (patients only): Perceived pain levels by patients will be measured with the Visual Analogue Pain Scale (VAS-P) consisting of a line of 10 centimeters divided into 10 points, each separated by 1 centimeter. 0 means complete absence of pain and 10 the maximum possible pain. The VAS-P is widely used in research and minimum clinically important differences pre-post interventions range from 9 millimeters [33] to 14 millimeters [34]. The VAS-P scale for pain will be applied before and after each intervention.
- Well-being: Well-being will be measured by the Well-being Numerical Rating Scales (WB-NRSs) developed by Bonacchi et al [35]. The WB-NRSs consist of a line divided into numbers from 1 to 10. 1 indicates a state of absolute distress and 10 a state of absolute well-being. The WB-NRSs uses 5 scales to measure well-being in 5 dimensions: physical, psychological, spiritual, relational, and general. The WB-NRSs will be applied before and after each intervention.

#### Sociodemographic and Basic Medical Data

To establish intergroup homogeneity, the following data will be collected from the patient’s medical history: age, primary diagnosis at the time of admission, triage classification (3 to 5), and the presence of a caregiver or companion at the time of admission to the emergency room.

#### Participant Timeline

**Figure 1:**
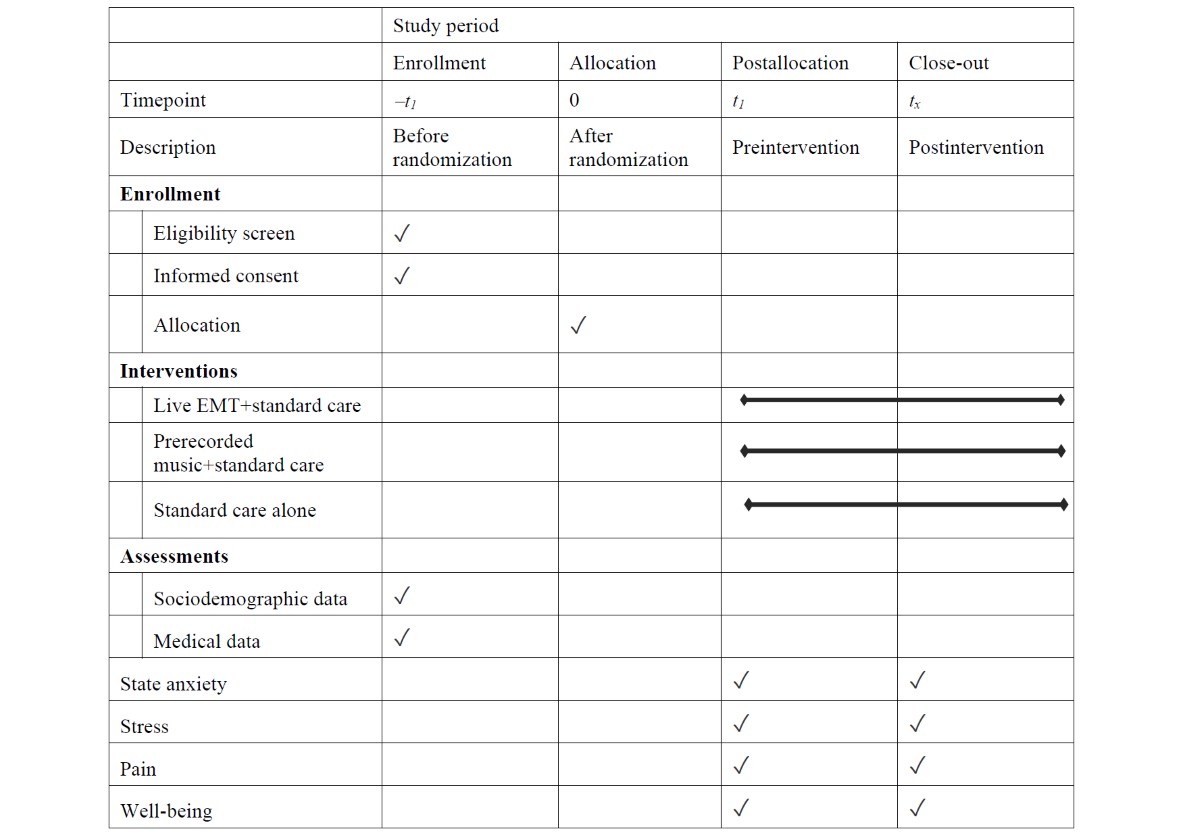
Participant timeline. EMT: Environmental Music Therapy.

Details of the participant timeline are listed in Figure 1.

### Intervention Protocol

#### Environmental Music Therapy: Intervention Group 1

Environmental Music Therapy is a music therapy technique that uses live music to modulate the potentially stressful effects of the environment toward an atmosphere that provides a sense of comfort and security. For this study, the intervention was adapted based on the 4 steps of EMT described by Rossetti [36]: assessment of the environment, initiation of the intervention, development of the intervention, and closure.

1. Evaluation of the environment: Before starting the intervention, the music therapist will observe the emergency waiting room for a couple of minutes to be aware of the number of people present and the content, volume, or timbre of the current auditory environment. In addition, it will be detected if, for example, someone shows signs of acute pain or signs of restlessness or anxiety. Assessing the environment also includes finding an optimal place for the music to be heard evenly throughout the room, without disturbing the movements and flow of patients, caregivers, and staff.
2. Beginning of the intervention: Before starting EMT, the music therapist will introduce him or herself to the patients and caregivers present and explain the role and purpose of the music. Patients and caregivers will be informed that they can participate in listening to the music in every way that feels comfortable for them, for example by just continuing what they are currently doing, by breathing slowly with the music, or by imagining themselves being in a safe place.
3. Development of the intervention: The instruments used will be an acoustic guitar (Yamaha C-40), an ocean drum (a drum with a double skin on which small metal balls roll imitating the sound of the waves), and a Samafon (an instrument consisting of aluminum tubes of different sizes that are struck with a soft mallet producing long-lasting single or combined tones). The different instruments and timbres will be introduced gradually. The music will consist of improvised instrumental music at a slow to moderate tempo, using free-flowing melodies, and simple chord progressions, focusing on creating moments of tension and resolution. The music therapist will also use his or her voice to accompany the music but without any lyrics or words. While the musical characteristics are aimed to remain consistent during the intervention, their selection, order, and content, will be adapted according to a constant re-evaluation of the environment.
4. Closing: after 20 minutes, the music therapist will gradually decrease the volume until the music stops.

#### Prerecorded Music: Intervention Group 2

The prerecorded music will consist of a recording including the same musical features and musical instruments as the live music. The recording will be made by the same music therapists who will perform the EMT intervention. To playback the music, a Bluetooth speaker (JBL Go 3) will be placed centrally in the ED waiting room and handled by the music therapists. The recorded music will last 20 minutes and the music therapists will remain present at the waiting room but will not engage in any further activities.

#### Standard Care Only: Control Group

The control group will receive standard care in the emergency room waiting room as provided by each hospital, but without being exposed to EMT or prerecorded music. However, the music therapist will remain present in the waiting room but will not engage in any activities. The control condition will last for 20 minutes.

### Data Collection and Storage

The data will be collected by a research assistant in each hospital and stored in a REDCap (Research Electronic Data Capture; Vanderbilt University) database to maintain confidentiality and data safety. During the data collection process, the database will be audited to identify the presence of inconsistent or atypical data. At the end of the data collection, a random selection of the data of 10% of the participants will be made to verify the paper version of the questionnaires against the electronic database.

### Potential Harms and Adverse Events

Any potential harm or adverse events related to the intervention will be recorded and communicated to the ethics committee according to the hospital site’s guidelines.

### Data Analysis

Regarding the sociodemographic and medical data, a descriptive analysis will be carried out. For continuous variables and according to the nature of the data, measures of central tendency and interquartile range or mean and SD will be used, and evaluated with the Shapiro-Wilk test. Categorical variables will be analyzed and summarized using frequencies. Association tests of categorical variables will be evaluated using the chi-square statistic or Fisher exact test in the case of tables with cells with expected values less than 5. For the comparison of measures of central tendency in the cases of quantitative variables, tests such as the Student *t* test or sum of Wilcoxon or Kruskal–Wallis ranges will be performed, depending on the presence or not of normality in the distribution of the variables. A statistical significance value of 5% will be used for all hypothesis test cases.

The analysis regarding the effect of the interventions will be carried out separately by type of participant (patient or caregiver). The effect on state anxiety, stress, pain (only patients), and well-being will be evaluated using 3×2 factorial ANOVA. Then a post hoc analysis will be performed using the Student *t* test and Bonferroni adjustment. A multiple linear regression analysis will be carried out to evaluate the variables (state anxiety, age, sex, type of participant, waiting time, and reason for consultation) that modify the effect of the interventions on the state anxiety, stress, pain, and well-being levels. Only data from patients and caregivers who will complete both baseline and postintervention questionnaires will be analyzed. However, to avoid bias we will assess if the distribution of participants who did not fill out the postintervention questionnaires is similar among the groups.

### Ethical Considerations

This study was approved by the Research Ethics Committee of the Fundación Universitaria Sanitas (CEIFUS 1779-24, date of approval: June 18, 2024). All participants and 2 additional witnesses will sign a written informed consent. Consent will be obtained from research assistants. This study was registered in ClinicalTrials.gov (NCT06510153).

## Results

Data collection for this study started on September 3, 2024, and as of the submission of the manuscript (November 2024), 216 patients and 134 caregivers have been enrolled. It is expected that the results will be available by May 2025.

## Discussion

### Anticipated Findings

This study seeks to investigate the effects of live EMT and prerecorded music on state anxiety, stress, pain, and well-being levels of patients and caregivers in the ED waiting room. Both patients and caregivers face multiple challenges while waiting to be attended to in the ED. Particularly, increased stress and anxiety levels have been found to be correlated with greater perceived barriers to care in ED patients [37]. Besides, long waiting times and noise can negatively impact not only patient satisfaction with care but also the satisfaction of those who accompany a patient [8,9]. In addition, anxiety levels in caregivers at ED discharge are correlated to their satisfaction with the ED visit [38].

While recorded music has previously been used in the waiting room to reduce distress and anxiety [39-41], we have not found studies that use live music or compare EMT and prerecorded music in ED waiting rooms. Furthermore, a recent meta-analysis on music in waiting rooms stresses the need for research with robust designs [20]. In this study, the multicenter approach, randomization of intervention days, and adequate sample size calculation for both patients and caregivers should help improve methodological soundness. While in this study we also use measures focusing on reducing mental health challenges (STAI-6, VAS-S), we actively seek to understand music’s role in improving well-being (WB-NRSs). The integration of both patients and caregivers into the intervention is in line with person-centered and humanized care models in Colombia [42].

### Potential Limitations

Several potential limitations have been identified for this study. First, participants may be called for treatment or medical exams after filling out the baseline questionnaires but before the end of the intervention, not all participants will fill out the postintervention questionnaires. Thus, only patients and caregivers who complete both baseline and postintervention questionnaires will be analyzed. Second, as data collectors need to be aware of who has already filled out the baseline questionnaires but might not be available anymore at the end of the interventions, blinded data collection is not feasible. However, we estimate expectancy effects to be low, as all questionnaires are self-rated and the data collector is not interfering with the filling out the forms. In addition, music therapists will be present during all 3 conditions to mitigate any potential effects due to their presence but do not interfere with either recruitment or data collection. However, in a future study, scales such as the Credibility and Expectancy Scale [43] could be applied to further mitigate expectancy effects in nonblinded participants. Fourth, while music therapists pretested the live music and prerecorded music to ensure similar volume and sound distribution in the ED waiting areas, no formal sound level measurements were applied. This could have resulted in slightly different sound experiences of participants and could be implemented in a future study to control for this variable. Third, anxiety, stress, pain, and well-being are influenced by multiple factors. In addition, both hospitals are in different cities and regions in the country. Thus, cultural and social factors may further influence the outcomes. While the pragmatic nature of this RCT acknowledges such complexity, results should nevertheless be interpreted with caution as not all confounding variables can be controlled.

### Conclusions

This research proposal seeks to advance knowledge on the role of music in improving mental health and pain in the ED waiting room. To our knowledge, it is the first multicenter RCT on music therapy in the wider field of emergency care in Colombia.

## Appendix

Multimedia Appendix 1 SPIRIT checklist.

## Abbreviations

ED: emergency department
EMT: Environmental Music Therapy
ESI: Emergency Severity Index
RCT: randomized clinical trial
REDCap: Research Electronic Data Capture
SPIRIT: Standard Protocol Items: Recommendations for Interventional Trials
STAI-6: 6-item State-Trait Anxiety Inventory
WB-NRS: Well-being Numerical Rating Scale
VAS-P: Visual Analog Pain Scale
VAS-S: Visual Analogue Stress Scale

## Data Availability

All data will be made available upon request by the corresponding author.
