# Correction: Effect of Live Environmental Music Therapy and Prerecorded Music on State Anxiety, Stress, Pain, and Well-Being Levels of Patients and Caregivers in the Emergency Department Waiting Room: Protocol for a Multicenter Randomized Clinical Trial

**DOI:** 10.2196/80077

**Published:** 2025-08-11

**Authors:** Angélica Hernández, Ana María Díaz, Ornella Fiorillo Moreno, Raúl Suárez, Moshé Amarillo, Ana María Moreno, Bryan Alonso Ríos Suarez, Lina Marcela Gómez González, Guiselle Alexandra Cristancho Olaya, Mark Ettenberger

**Affiliations:** 1 Clínica Keralty Ibagué Clínica Colsanitas Ibagué, Tolima Colombia; 2 SONO - Centro de Musicoterapia Bogotá Colombia; 3 Clínica Iberoamérica Clínica Colsanitas Barranquilla Colombia; 4 Research Department SONO - Centro de Musicoterapia Bogotá Colombia

In “Effect of Live Environmental Music Therapy and Prerecorded Music on State Anxiety, Stress, Pain, and Well-Being Levels of Patients and Caregivers in the Emergency Department Waiting Room: Protocol for a Multicenter Randomized Clinical Trial” (JMIR Res Protoc 2025;14:e69131), the authors noted one error:

In the originally published paper, reference 27 appeared as:

Reger DL, Little CA, Rheingold AL, Lam M, Liable-Sands LM, Rhagitan B, et al. A synthetic, structural, magnetic, and spectral study of several [Fe[tris(pyrazolyl)methane]2](BF4)2 complexes: observation of an unusual spin-state crossover. Inorg Chem. 2001;40(7):1508-1520. doi: 10.1021/ic001102t. Medline: 11261958

This has been corrected to:

Chlan L, Savik K, Weinert C. Development of a shortened state anxiety scale from the Spielberger State-Trait Anxiety Inventory (STAI) for patients receiving mechanical ventilatory support. J Nurs Meas. 2003 Winter;11(3):283-93. doi: 10.1891/jnum.11.3.283.61269. PMID: 15633782

The correction will appear in the online version of the paper on the JMIR Publications website, together with the publication of this correction notice. Because this was made after submission to PubMed, PubMed Central, and other full-text repositories, the corrected article has also been resubmitted to those repositories.

